# Comparison of epiphytic and intestinal bacterial communities in freshwater snails (*Bellamya aeruginosa*) living on submerged plants

**DOI:** 10.7717/peerj.14318

**Published:** 2022-11-03

**Authors:** Fucai Liu, Kejun Li

**Affiliations:** College of Marine Ecology and Environment, Shanghai Ocean University, Shanghai, China

**Keywords:** Bacterial community, Bacterial correlation, Bacterial diversity, Epiphytic bacteria, Intestinal bacteria

## Abstract

The combination of submerged plants and snails can combat eutrophication of freshwater systems by suppressing algal growth and assimilating nutrients. By consuming epiphytes, snails can benefit the growth of submerged plants. However, the efficiency of this phytoremediation strategy may depend on the microbes associated with the plants and snails. In this study, we compared the epiphytic bacterial communities on submerged plants (*Vallisneria natans* and *Cabomba caroliniana*) and intestinal bacterial communities of a snail, *Bellamya aeruginosa,* found on these plants using 16S rRNA gene sequencing. Epiphytic bacterial communities were similar between the two plant species and snails shared a high proportion of snail intestinal bacterial OTUs (75%) and genera (85%) with plants they grazed on. However, significant variations of Bray-Curtis distances differentiated epiphytic and intestinal bacterial communities. In addition, between the top 50 genera shared by intestinal and epiphytic bacterial communities, more Spearman correlations were detected within bacterial communities associated with snails than between communities associated with plants (190 *vs*. 143), and the correlations in epiphytic bacterial networks were more concentrated on certain genera, indicating they possessed distinct bacterial networks. This suggests the bacterial communities associated with snails do not depend strongly on the plant they graze on, which may be important for better understanding the role of snails in aquatic eco-restoration.

## Introduction

Surface water pollution with nitrogen and phosphorus has increased in many regions worldwide (Yao, You & Liu, 2016; Wang et al., 2019). Most strategies developed to manage this eutrophication have focused on limiting nutrient loading to receiving waters; however, phytoremediation also shows promise (Guittonny-Philippe et al., 2014; Ning et al., 2014; Li et al., 2018). For example, actively growing submerged plants assimilate nutrients, release allelochemicals that control planktonic algae (Li et al., 2021), and improve water clarity. In recent years, submerged plants have been used to remediate a series of reclaimed wastewater lakes (Li et al., 2014) and freshwater lakes (Fan et al., 2018; Ge, Zhang & Yang, 2018; Liu, Liu & Xing, 2021). However, excess nutrients in the water column can led to excessive epiphytes which contribute to shading that limits the growth of submerged plant, and snails can enhance submerged plant growth by removing epiphytes attached to plant surfaces (Yang et al., 2020; Zhi et al., 2020). For example, Zhang et al. (2020) reported that snails (*Bellamya aeruginosa*) were beneficial to the growth of the submerged plant *Vallisneria natans* and Ren et al. (2022) reported that the combination of submerged plants and snails can improve water clarity.

Bacteria play key roles in nutrient cycling in water (Salazar & Sunagawa, 2017) and in pollutant removal (Xu et al., 2018). Bacteria attached to the surfaces of submerged plants have displayed great potential as a means of coping with pollution (Zhang et al., 2016; Yan et al., 2018; Geng et al., 2019; Xia et al., 2020). Epiphytic bacteria on the surfaces of submerged plants have higher species richness than planktonic bacteria in various freshwater lakes, and a marked divergence in the community structure exists between epiphytic bacteria and bacterioplankton (He, Ren & Wu, 2014; Xian et al., 2020; Yu et al., 2022). Lachnit et al. (2009) found that epiphytic bacterial communities differed less between regions than between host species; particular, closely related host species. By contrast, Xia et al. (2020) reported that epiphytic bacterial communities among three submerged macrophytes were not significantly different. In snails, intestinal bacteria play important roles in the immunity, development, nutrition, and digestion of their hosts. In particular, cellulolytic bacteria in the snail microbiome help their host digest plant fiber (Hu et al., 2018). Li et al. (2019) compared the bacterial communities in the buccal mass, stomach, and intestine of the snail *Pomacea canaliculata* and found that the bacterial diversity was high in the intestine and low in the buccal mass. Zhang et al. (2020) reported that snails (*B*. *aeruginosa*) could decrease the richness and diversity of the epiphytic microbial community on the plants. However, the comparison of epiphytic bacterial communities on submerged plants and intestinal bacterial communities in snails living on submerged plants has not been reported.

Given that snails scrape epiphytic bacteria as food, epiphytic bacterial communities might impact snail intestinal bacterial communities. A better understanding of this interaction may improve aquatic eco-restoration programs and our understanding of plant-microbe-snail relationships. In this study, we compared intestinal bacteria from the freshwater snail *B. aeruginosa* living on submerged plants (*V. natan*s and *Cabomba caroliniana*) with epiphytic bacteria and found that the snail intestinal bacterial communities do not depend strongly on the plant they graze on.

## Material and Methods

### Sample collection

We collected samples from one small and shallow freshwater lake in Shanghai, China. This lake covers an area of approximately 60,000 m^2^, with an average depth of approximately 2 m. The floor zone of this lake was covered by clusters of submerged plants (*V. natans* and *C. caroliniana*) with snails (*B. aeruginosa*) living on their leaves. Lake water, submerged plant leaves, and snails on leaves were sampled from each of two adjacent clusters of submerged plants (the two mentioned species and approximately 1 m^2^ for each species, E121.8980°N 30.8850°) three times in November, 2021 at 1 week intervals. In total, three planktonic samples, six epiphytic samples, and six intestinal samples from 36 snail individuals (1.1 ± 0.2 g) were collected.

On each sampling date, we collected 500 ml of lake water from each plant cluster and mixed them. Immediately after water sampling and mixing, 500 ml of water sample were filtered through a 5 µm pore mixed cellulose ester (MCE) filter (Xinya, Shanghai, China) to remove suspended sediment and planktonic algae (Zhao et al., 2017; Liu et al., 2019) and then through a 0.22 µm pore MCE filter (F513134; Sangon Biotech, Shanghai, China) to collect bacterial samples. Collected bacterial samples on the filters were placed in sterile centrifuge tube and stored at −20 °C for subsequent analysis.

The remaining water samples were used to determine total nitrogen (3.75 ± 1.52 mg l^−1^), total phosphorus (0.15 ± 0.08 mg l^−1^), ammonia nitrogen content (0.80 ± 0.87 mg l^−1^), and the permanganate index (7.51 ± 0.57 mg l^−1^). We also determined the dissolved oxygen content (5.4 ± 1.9 mg l^−1^), water temperature (13.8 ± 1.9 °C), pH (7.49 ± 0.23), and salinity (0.70 ± 0.07 ‰) *in situ*. Detailed methods and data are provided in the Supporting Information (Table S1) as the environmental background.

We also collected 5 g of submerged plant leaves from each plant cluster on each sampling date. After sampling, the leaves were transferred into 50 ml sterile centrifuge tubes containing 30 ml of sterile ultrapure water from a water purification system (Direct –Q5 UV; Merck Millipore, Darmstadt, Germany) and treated with ultrasonic waves for 5 min using an ultrasonic cleaner (JP-020S; Skymen, Shenzhen, China) to release the epiphytic bacteria from the leaves (Xia et al., 2020; Wu et al., 2021). The eluents were collected and centrifuged at 10,000×*g* for 5 min to collect the bacterial pellets. The bacteria samples were stored at −20 °C for subsequent analysis.

In addition, we collected six snail individuals from the leaves in each plant cluster on each sampling date. The weight of each individual snail is provided in Table S2. The digestive tract from the stomach to the anus, excluding the stomach, was carefully isolated from snails under aseptic conditions (Hu et al., 2018; Li et al., 2019; Hu et al., 2021). The intestinal content of an individual snail was insufficient for the sequencing analysis; therefore, we collected the intestinal contents from the six snails, pooled them into a sterile centrifuge tube, and mixed them evenly. Intestinal samples were stored at −20 °C for subsequent analysis. All experiments involving animals were performed in accordance with the protocols approved by the Animal Ethics Committee of Shanghai Ocean University (Approval ID: SHOU-DW-2020-059).

### Amplicon sequencing

Bacterial metagenomic DNA in each bacterial sample was extracted using an E.Z.N.A. soil DNA Kit (Omega Bio-tek, Norcross, GA, USA). Using the primer pairs 338F and 806R (Srinivasan et al., 2012) with sequencing barcodes, the V3 −V4 region of the bacterial 16S rRNA gene was amplified from the metagenomic DNA using an ABI GeneAmp 9700 PCR thermocycler (Applied Biosystems, Carlsbad, CA, USA). The PCR product was purified using an AxyPrep DNA Gel Extraction Kit (Axygen Biosciences, Union City, CA, USA) and quantified using a Quantus fluorometer (Promega, Madison, WI, USA). Purified amplicons were paired-end sequenced (2 × 300 bp) on an Illumina MiSeq platform (Illumina, San Diego, CA, USA) by a commercial company (Majorbio, Shanghai, China). The raw 16S rRNA gene sequencing reads were quality-filtered using fastp (Chen et al., 2018) and merged using FLASh (Magoč & Salzberg, 2011).

### Bioinformatics and statistical analysis

Operational taxonomic units (OTUs) with a 97% similarity cutoff were clustered using Uparse (Edgar, 2013). The taxonomy of each OTU representative sequence was analyzed using RDP Classifier (Wang et al., 2007) against the Silva v 138 16S rRNA database (Quast et al., 2013). Alpha diversity indexes were calculated using Mothur (Schloss et al., 2009) and beta diversity (Bray-Curtis) distances were calculated using Qiime2 (Caporaso et al., 2010; Bolyen et al., 2019). We submitted the raw gene sequences obtained in this study to the NCBI SRA database (https://submit.ncbi.nlm.nih.gov/subs/sra/) under the accession number PRJNA793358.

Statistical analysis was carried out using the R software (version 3.3.1, the R Foundation, Vienna, Austria). Diversity indexes and relative abundances of dominant bacterial phyla between sample groups were compared using Student’s *t*-test and Wilcoxon rank-sum test (R stats package). The differences in community structures were detected using non-metric multidimensional scaling analysis, analysis of similarities, and hierarchical clustering community heatmaps (R vegan package). The numbers of bacterial OTUs shared by sample groups were displayed using Venn diagrams (R Venndiagram package). In addition, bacterial network analysis was carried out using the Networkx package (https://networkx.org/).

## Results

### Bacterial diversity

Using Illumina MiSeq sequencing, we obtained 762,385 clean sequence reads with an average length of 415 base pairs from the bacterial 16S rRNA gene after quality filtering. After removing chloroplast sequences and conducting normalization, 27,566 sequences from each sample remained for subsequent analysis. After clustering, we obtained a total of 4020 OTUs. The OTU table and the alpha diversity indexes are provided in Supporting Information (Tables S3 and S4). For all gene libraries, Good’s coverage values exceeded 97.8%.

Habitats not plant host species determined alpha diversity and bacterial communities in this study. Student’s *t*-test analyses of the Sobs, Shannon, Simpson, Ace, and Chao indexes showed no significant variation in bacterial alpha diversity between the epiphytic samples (the *P* values were 0.22, 0.99, 0.56, 0.38, and 0.40, respectively) or between the intestinal samples (the *P* values were 0.95, 0.87, 0.72, 0.45, and 0.37, respectively) from the two submerged plant species. In contrast, significant differences in the Ace and Chao index values were found between the planktonic sample group and each of the other sample groups (*P* = 0.001), and significant variation of the Chao index values were detected between the epiphytic and intestinal samples (*P* = 0.026).

**Figure 1:**
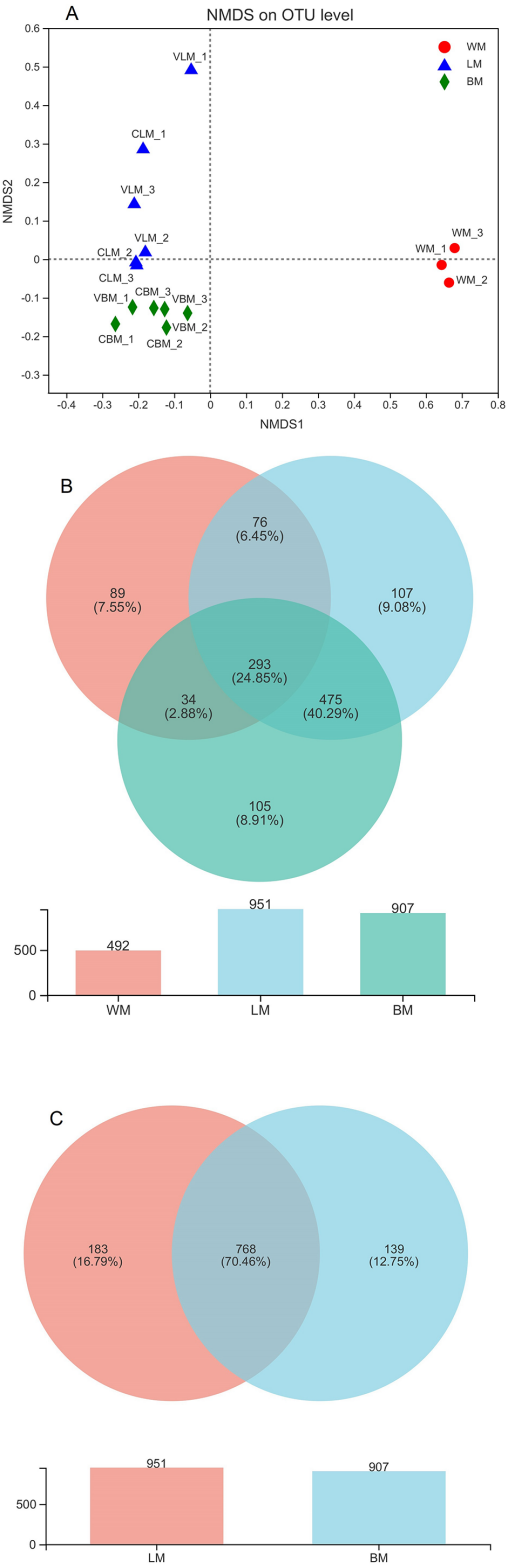
Non-metric multidimensional scaling analysis and Venn diagrams. (A) Non-metric multidimensional scaling analysis (stress = 0.05) showing significant variations among the three habitats. (B) Venn diagram showing the number of core bacterial operational taxonomic units (OTUs) in bacterial communities from the three habitats, and a histogram showing the number of bacterial OTUs. (C) Venn diagram showing the number of core bacterial genera in the epiphytic and intestinal bacterial communities, and a histogram showing the number of bacterial genera. WM, LM, and BM stand for planktonic, epiphytic, and snail intestinal bacterial communities, respectively.

Analysis of similarities also showed no significant variations in bacterial beta diversity between the epiphytic samples (*R* = 0.037, *P* = 0.305) or between the intestinal samples (R = −0.2593, *P* = 0.82) from the two submerged plant species. However, non-metric multidimensional scaling analysis showed significant variations between the planktonic sample group and the other sample groups (Fig. 1A). The results of analysis of similarities also confirmed the variations between planktonic and epiphytic (or intestinal) samples (*R* = 1 and *P* = 0.015). Further analysis of similarities revealed significant variations between the epiphytic samples and intestinal samples (*R* = 0.4426, *P* = 0.001). These results were consistent with the analysis of the alpha diversity. Therefore, we mixed the samples from both plant species in subsequent analyses.

### Core genera

The bacterial genera were highly similar between the epiphytic and snail intestinal bacterial communities. A Venn diagram revealed that 380 core OTUs were shared by the three sample groups, representing 57% of all sequences involved in the analysis. In addition, another 1,772 OTUs were shared by the epiphytic and intestinal samples (Fig. 1B). In total, 2,152 core OTUs accounted for 70% and 75% of the epiphytic and intestinal OTUs, respectively. At the genus level, the Venn diagram showed that 951 genera were found in the epiphytic samples and 907 genera were found in the intestinal samples, with 768 genera being shared between them (Fig. 1C). The shared genera accounted for 81% and 85% of genera in the epiphytic and intestinal samples, respectively.

### Community structures

**Figure 2:**
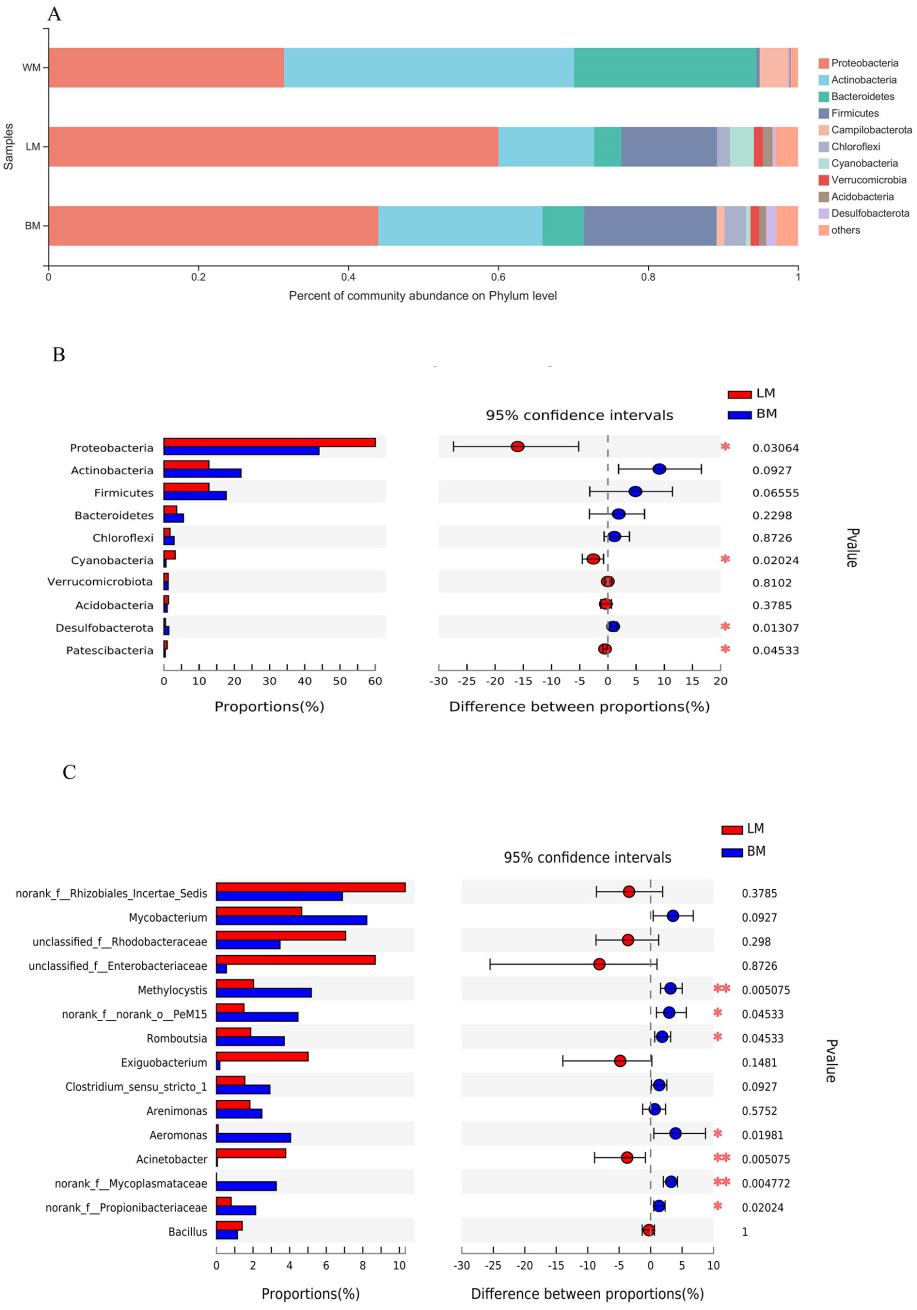
Bacterial community structures and Wilcoxon rank-sum test results. (A) Bacterial community structures in the three habitats at the phylum level. (B) Wilcoxon rank-sum test results of the 10 most dominant phyla between the epiphytic and intestinal bacteria samples. (C) Wilcoxon rank-sum test results of the 15 most dominant genera between the epiphytic and intestinal bacteria samples.

The dominant bacterial phyla in the planktonic samples were Actinobacteria, Proteobacteria, and Bacteroidetes, with relative abundances of 39%, 32%, and 24%, respectively, whereas the dominant bacterial phyla in the epiphytic and intestinal samples were Proteobacteria, Actinobacteria, and Firmicutes, with relative abundances of 60%, 18%, and 18%, respectively, in the epiphytic samples; and 44%, 22%, and 18%, respectively, in the intestinal samples (Fig. 2A). Relative abundances of four of the top ten phyla differed significantly between the epiphytic and intestinal samples (Fig. 2B). The abundances of Proteobacteria, the top phylum, and Cyanobacteria and Patescibacteria were significantly higher in the epiphytic samples, whereas Desulfobacterota was significantly more abundant in the intestinal samples. For the other six phyla, the mean values of the relative abundances of Actinobacteria, Firmicutes, Bacteroidetes, and Chloroflexi were higher in the intestinal samples and those of Verrucomicrobiota and Acidobacteria were higher in the epiphytic samples; however, the variations were not significant (*P* > 0.05).

**Figure 3:**
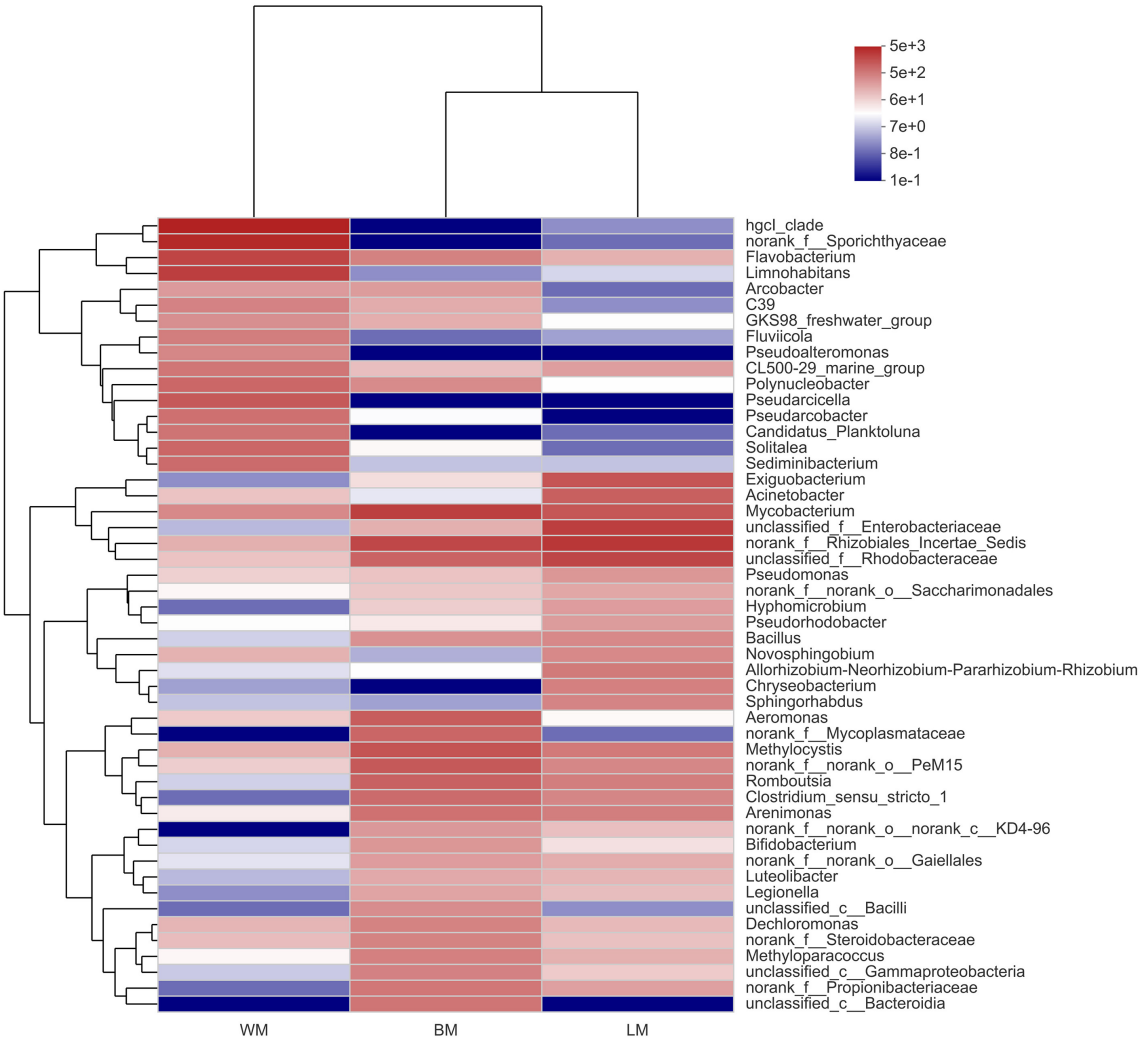
Community heatmap showing the abundances of the top 50 bacterial genera and the Bray-Curtis distances between sample groups. WM, LM, and BM stand for planktonic, epiphytic, and snail intestinal bacterial communities, respectively.

At the genus level, the bacterial communities in the three habitats were dominated by various phylogenetic lineages (Fig. 3 and Fig. S1). Not considering genera with relative abundances <5%, planktonic bacteria were dominated by hgcl_clade (17%), norank_f__Sporichthyaceae (14%), *Limnohabitans* (8.7%), and *Flavobacterium* (7.5%), whereas norank_f__Rhizobiales_Incertae_Sedis (10%), unclassified_f__Enterobacteriaceae (8.7%), unclassified_f__Rhodobacteraceae (7.1%), and *Exiguobacterium* (5.0%) dominated the epiphytic bacteria. The intestinal bacterial community was dominated by *Mycobacterium* (8.2%), norank_f__Rhizobiales_Incertae_Sedis (6.9%), and *Methylocystis* (5.2%). In particular, many Cyanobacteria genera were highly abundant in the epiphytic samples; however, only unclassified_f__Phormidiaceae (44%), *Planktothrix* _NIVA-CYA_15 (22%), and *Cyanobium* _PCC-6307 (16%) dominated the intestinal Cyanobacteria communities and these Cyanobacteria genera were also highly abundant in the epiphytic samples (Fig. S2).

The relative abundances of seven of the top 15 genera differed significantly between the epiphytic and intestinal samples (Fig. 2C). Among these seven genera, six were more abundant in the intestinal samples and only *Acinetobacter* was more abundant in the epiphytic samples. In the eight genera without significant variations, only four could be classified: *Mycobacterium*, *Exiguobacterium*, *Arenimonas*, and *Bacillus*.

### Network analysis

**Figure 4:**
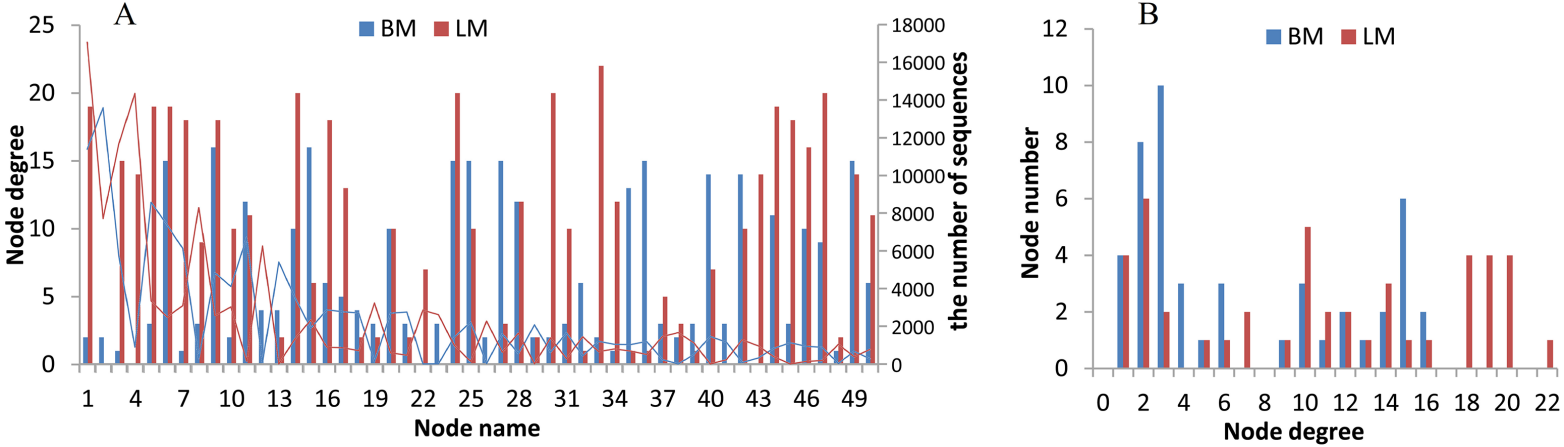
Bacterial network analysis results of the top 50 genera shared by epiphytic and intestinal bacterial communities. (A) Bars showing the node degrees (the number of significantly correlated genera) of the 50 nodes (the top 50 core genera, ranked from 1 to 50 in the order of relative abundance) and the lines showing the sequence number of each genus. (B) The node number (the number of bacterial genera) of each node degree level.

The two niches possessed distinct bacterial networks, and the correlations in the snail intestines were dispersed across the bacterial network, but the correlations in the epiphytic network were more concentrated on certain genera. Based on the Spearman correlation coefficients among bacterial genera, we compared the bacterial networks between the epiphytic and intestinal bacterial communities. For the comparison of two habitats, we analyzed the top 50 genera (provided in Table S5) from 768 genera shared by the epiphytic and intestinal bacterial communities. The top 50 genera represented 57% and 70% of sequences from the epiphytic and snail intestinal bacterial communities, respectively. Under the criteria of the absolute values of *R* ≥ 0.5 and *P* < 0.05, 143 and 190 significant correlations were found in the epiphytic and intestinal bacterial communities, respectively (Fig. 4). There were also some genera, for example, nodes 9 (*Clostridium* _*sensu* _*stricto* _1), 11 (*Aeromonas*), 28 (*Pseudomonas*), 42 (*Trichococcus*), and 49 (norank), which possessed similar and high node degree levels in both sample groups (see also Table S5), indicating their important roles in the two different environments. Some obvious differences were also observed. The top genus (node 1 in Fig. 4A) correlated significantly with 19 genera (node degree 19) in the epiphytic bacterial communities; however, this genus had only two significantly correlated genera (node degree 2) in the intestinal bacterial communities. Node 33 (*Legionella*) was closely related to 22 genera in the epiphytic samples, but to only 2 genera in the intestinal samples. By contrast, nodes 27 and 36 were closely related to 15 genera in the intestinal samples, but to only three and one genera in the epiphytic samples, respectively. In the top six node degrees (node degrees 17–22 in Fig. 4B), no genera were found in the intestinal bacterial communities; whereas, in the epiphytic bacterial communities, there was one genus that correlated significantly with 22 genera (see also node 33 in Fig. 4A), and four genera that correlated significantly with each of node degrees 18–20.

## Discussion

Submerged plants provide valuable ecosystem services in shallow freshwater lakes (Ge, Zhang & Yang, 2018). Epiphytic microorganisms on the surfaces of submerged plants have great potential to mitigate the water pollution (Geng et al., 2019). Studies have reported that the community structure of the epiphytic and planktonic bacterial communities differ (He, Ren & Wu, 2014; Xian et al., 2020; Yu et al., 2022). In the present study, we also detected significant differences between the planktonic and epiphytic bacterial communities, with higher alpha and beta diversities in the epiphytic bacterial communities than the surrounding planktonic bacteria. Liu, Liu & Xing (2021) reported that the flat-leaf type and needle-leaf type of submerged plants possessed different functional traits in eutrophic shallow lakes, which affected their ecological functions. However, we did not find significant variations of alpha and beta diversities in the epiphytic bacterial samples between the flat-leaf type (*V*. *natan* s) and needle-leaf type (*C*. *caroliniana*) of plants evaluated in our study, which agreed with the results from Xia et al. (2020). The structure of the epiphytic bacterial communities also depends on the environmental conditions (Bao et al., 2020; Comba González et al., 2021) and the growth states and even decompositions of the hosts (Han et al., 2019); thus, it is not a simple reflection of the host species.

Worldwide, snails play an important role in nutrient cycling by consuming plant and epiphytic biomass in freshwater ecosystems, and their gut bacteria contribute greatly to their nutrition and digestion (Hu et al., 2018). Only limited studies have focused on the intestinal microbial communities in freshwater snails. Hu et al. (2018) reported that the bacterial phyla in the gut of *Radix auricularia* were predominantly Proteobacteria and Cyanobacteria. For *P. trivolvis* raised on herbivorous feed, the predominant gut microbiota were Proteobacteria and Bacteroidetes, whereas snails that were fed with non-herbivorous feed contained predominantly Proteobacteria (Hu et al., 2021). However, to date, few studies have focused on the comparison of intestinal microbial communities in freshwater snails with surrounding epiphytic microbial communities. In the present study, Proteobacteria also dominated both the intestinal and epiphytic bacterial communities, followed by Actinobacteria and Firmicutes; however, higher relative abundances of Proteobacteria were found in the epiphytic bacterial communities in comparison with the snail intestinal bacterial communities, and their community structures differed markedly. Additionally, no significant variations were found in the snail intestinal bacterial communities from the two species of submerged plant with the analogous epiphytic bacterial communities, whereas the similarity of the intestinal bacterial communities in snails living on submerged plants with different epiphytic bacterial communities remains unclear. Although significant differences in the intestinal bacterial communities in *Cipangopaludina chinensis* on *V*. *natans* and *Elodea Canadensis* from different ecologically restored lakes were reported (Li, 2012), further insights into these snail intestinal bacterial communities under different epiphytic environments and their ecological functions will aid aquatic ecological restoration and pollution removal.

In the present study, the high proportions of intestinal bacterial OTUs and genera were shared by epiphytic samples; however, only a low proportion of bacterial OTUs were shared by the planktonic samples, indicating a higher similarity between epiphytic and intestinal bacterial communities. Yang et al. (2020) studied the effects of herbivorous snails (*R*. *swinhoei*) on the biomass of epiphytic algae and the growth of the submerged macrophyte, *V*. *denseserrulata*, under contrasting nitrogen and phosphorus loadings. The results showed that when snails were present, the biomass of epiphytic algae decreased significantly and the growth of *V. denseserrulata* increased significantly, with a more pronounced effect in the nutrient-rich treatment group (Yang et al., 2020). Zhang et al. (2020) investigated the impact of *B. aeruginosa* at different densities on the submerged plant *V. natans* and concluded that snails could decrease the richness and diversity of the epiphytic microbial community. These snail species scrape off the epiphytic biomass, promote nutrient cycling, and are widely used to maintain a clear water state (Li, 2012; Ren et al., 2022). The high proportion of bacterial OTUs shared by the intestinal and epiphytic bacterial communities indicated that they were likely to influence each other *via* the scraping and also possibly defecation of snails.

Some differences were found between the epiphytic and intestinal bacterial communities. For example, the abundances of *Acinetobacter* and *Aeromonas* varied significantly between the epiphytic and intestinal bacterial communities. *Acinetobacter* is a ubiquitous genus and is recognized as a potential pathogen of great concern to public health (Carvalheira, Silva & Teixeira, 2021). Its abundance was significantly higher in the epiphytic community in our study and in the gut of *R. auricularia* (Hu et al., 2018) and *P. trivolvis* (Hu et al., 2021). *Aeromonas* is a cellulolytic bacterial genus found in snails (Hu et al., 2018), whose abundance was significantly higher in the intestinal community in our study and it was dominant in the gut of most previously reported snails and *P. canaliculata* (Li et al., 2019). As established endosymbiotic bacteria, *Aeromonas* were found in both adult and juvenile *Potamopyrgus antipodarum* in Lake Sarah, suggesting that snails might inherit these bacteria or acquire them soon after birth (Takacs-Vesbach et al., 2016). Furthermore, the epiphytic and snail intestinal bacterial networks were distinct, indicating that different functional units exist in the two habits. The co-occurrences and interactions between bacterial taxa result in complex bacterial networks and the network analysis helps us to understand these interactions, as a core topic of microbial ecology (Coyte, Schluter & Foster, 2015). The nodes with significant correlations between each other are often treated as functional units in biological systems (Deng et al., 2012) and the high node levels were generally defined as key bacteria in bacterial communities (Chen et al., 2020; Li et al., 2020). Some genera, for example *Aeromonas*, possessed numerically similar and high node levels in both sample groups, indicating their important roles in the two different environments, whereas those nodes (genera) possessing high node levels only in one habit possibly play an important role in the unique ecological functions of the bacterial communities.

## Conclusions

In this study, we compared the epiphytic and intestinal bacterial communities in freshwater snails (*B*. *aeruginosa*) living on submerged plants (*V. natan* s and *C. caroliniana*). We did not detect significant variations of the epiphytic and intestinal bacterial communities between the two submerged plant species. Furthermore, a high proportion of snail intestinal bacterial OTUs and genera were shared by the epiphytic bacterial communities. However, some differences were also found between the epiphytic and intestinal bacterial communities. Community structures and bacterial networks varied significantly between the epiphytic and intestinal bacterial communities. For example, significantly lower Cyanobacteria abundances and fewer dominant Cyanobacteria genera were found in the intestinal bacterial communities. In addition, more bacterial correlations between the top 50 core genera were detected in the snail intestines, and the bacterial correlations in snail intestines were less concentrated on certain genera compared with those in the epiphytic bacterial communities. The results showed that the snail intestinal bacterial communities were obviously different to the epiphytic bacterial communities, despite the snails scraping off the epiphytic bacteria as food, and thus, the epiphytic bacteria might impact the intestinal bacterial communities in snails.

##  Supplemental Information

10.7717/peerj.14318/supp-1 Supplemental Information 1 The variations in relative abundance among bacterial taxa from the phylum level to the genus level (from the inside out) Linear discriminant analysis effect size (LEfSe) analysis (http://huttenhower.sph.harvard.edu/galaxy/) was used in this study.

10.7717/peerj.14318/supp-2 Supplemental Information 2 Community heatmap showing the relative abundances of all 60 Cyanobacteria genera found in this study

10.7717/peerj.14318/supp-3 Supplemental Information 3 Water quality assessment for the three sampling dates

10.7717/peerj.14318/supp-4 Supplemental Information 4 The weight of each individual sampled snail

10.7717/peerj.14318/supp-5 Supplemental Information 5 The OTU table

10.7717/peerj.14318/supp-6 Supplemental Information 6 Bacterial alpha diversity indexes

10.7717/peerj.14318/supp-7 Supplemental Information 7 The top 50 genera shared by the epiphytic and intestinal bacterial communities, as supporting information for Fig. 4

## Additional Information and Declarations

### DNA Deposition

The following information was supplied regarding the deposition of DNA sequences: The raw gene sequences obtained in this study are available at NCBI SRA: PRJNA793358.

### Data Availability

The following information was supplied regarding data availability: The raw data is available in the Supplemental Files.
