# Supplementary figures and images for: Comparison of epiphytic and intestinal bacterial communities in freshwater snails (Bellamya aeruginosa) living on submerged plants
Source: PeerJ. 2022 Nov 3;10:e14318. doi: 10.7717/peerj.14318 (PMC9637354; doi:10.7717/peerj.14318)

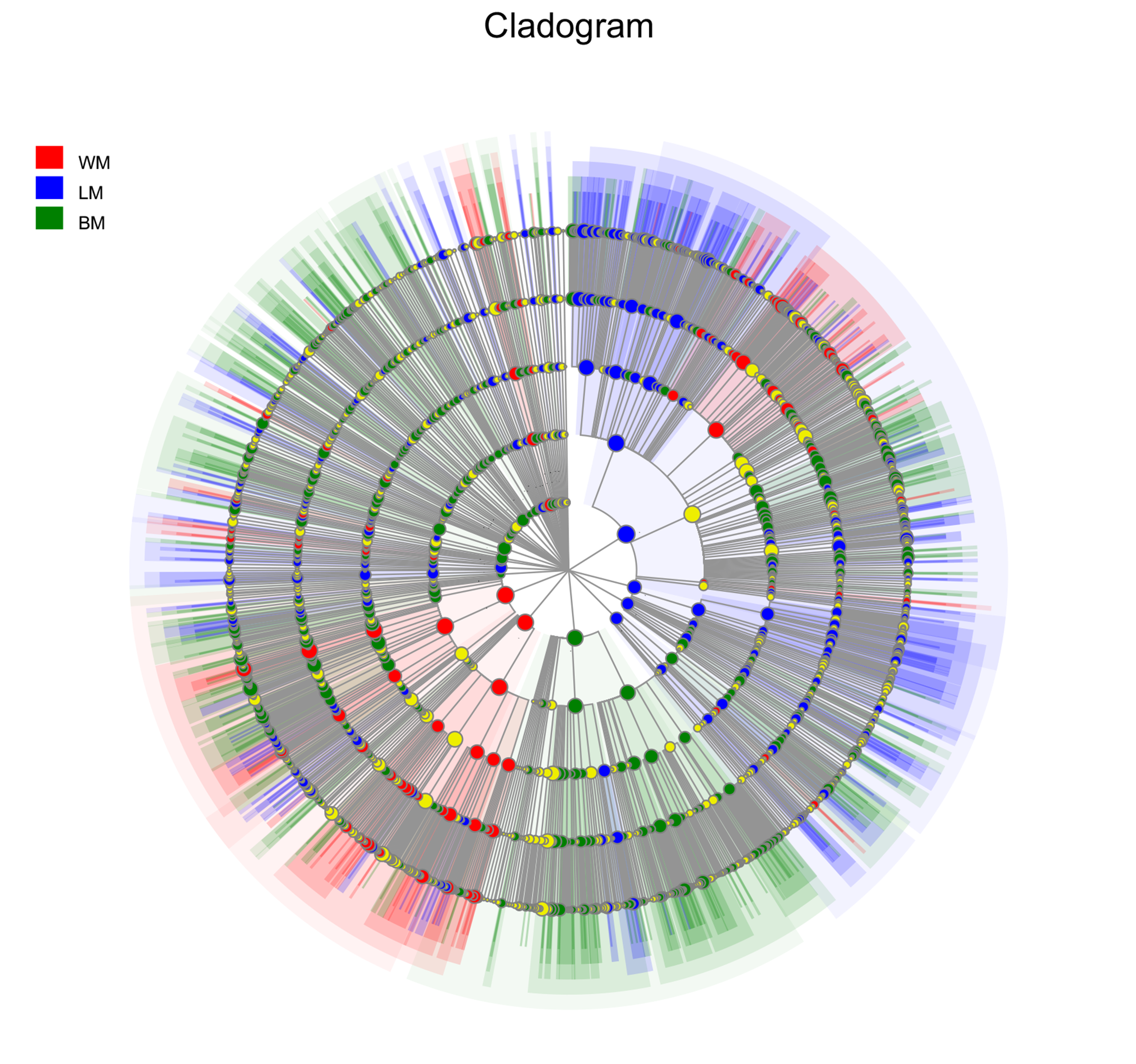

Supplement: Supplemental Information 1 — Linear discriminant analysis effect size (LEfSe) analysis (http://huttenhower.sph.harvard.edu/galaxy/) was used in this study. [file peerj-10-14318-s001.tif]

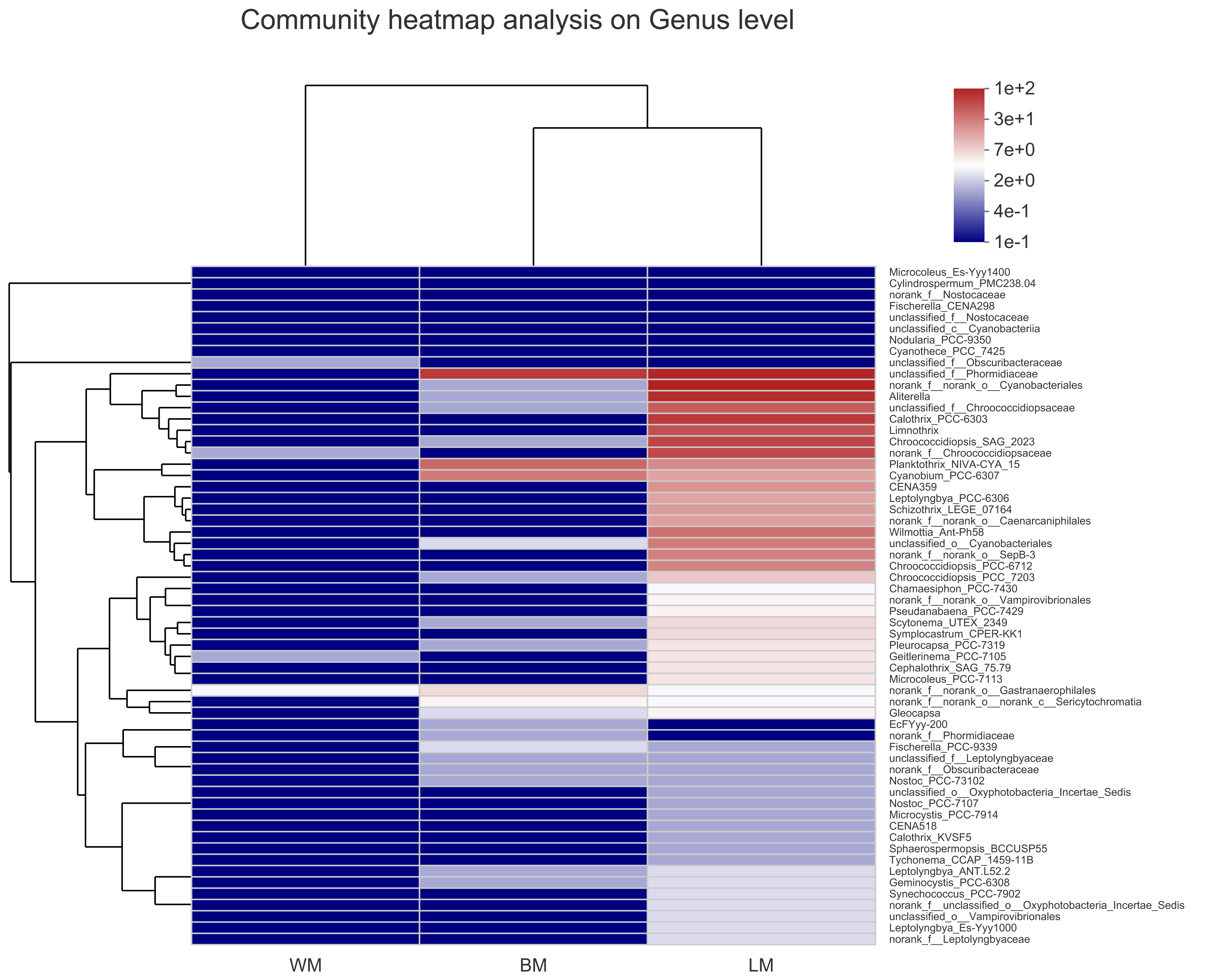

Supplement: Supplemental Information 2 [file peerj-10-14318-s002.tif]
